# Role of distance in the use of the Emergency Departments for dental conditions among older adults in Maryland

**DOI:** 10.1093/geroni/igaf122.2019

**Published:** 2025-12-31

**Authors:** Uma Kelekar, Shillpa Naavaal, Debasree Das Gupta, Sidney Turner

**Affiliations:** Marymount University, Arlington, Virginia, United States; Virginia Commonwealth University, Richmond, Virginia, United States; Utah State University, Logan, Utah, United States; Fors Marsh Group, Arlington, Virginia, United States

## Abstract

Disparities in access to oral health in the US are evident in the utilization of Emergency Departments (ED) for non-traumatic dental conditions (NTDCs). However, evidence regarding the role of distance in ED utilization for NTDCs, particularly among older adults (≥65) remains limited. To fill this gap, our objective was to compare how ED utilization among different age-groups varied based on the location of nearest EDs and community dental providers. We used State Emergency Department Database and American Dental Association provider data files for 2017-2021 for Maryland. Using negative binomial regressions, we estimated ED utilization rates for different age-groups as a function of two types of distances—1) patient zip codes and nearest EDs and 2) patient zip codes and dental providers, while controlling for confounders. Compared to other age-groups, older adults reported the lowest ED visits (296 per 100,000) and lived the farthest from the nearest ED. The regression results revealed no statistically significant differences in ED utilization rates for NTDCs across the different age-groups. Nevertheless, among older adults, the average NTDC utilization rate decreased as the distance to the nearest ED increased (β=-0.22; 95%CI: -0.31,-0.13), while it increased with greater distances to the nearest dental provider (β = 0.19; 95%CI: 0.08,0.30). Together, these results indicate that the 65+ are less likely to use emergency services for NTDCs when they are farther away from an ED, particularly if a dental provider is nearby. Thus, our findings highlight the complex interplay of access-based factors with perceived needs of dental care among the 65+.

